# Physical Factors Influencing Pleasant Touch during Tactile Exploration

**DOI:** 10.1371/journal.pone.0079085

**Published:** 2013-11-14

**Authors:** Anne Klöcker, Michael Wiertlewski, Vincent Théate, Vincent Hayward, Jean-Louis Thonnard

**Affiliations:** 1 Institute of Neuroscience, Université catholique de Louvain, Brussels, Belgium; 2 UPMC Univ Paris, Institut des Systèmes Intelligents et de Robotique, Paris, France; VU University Amsterdam, Netherlands

## Abstract

**Background:**

When scanning surfaces, humans perceive some of their physical attributes. These percepts are frequently accompanied by a sensation of (un)pleasantness. We therefore hypothesized that aspects of the mechanical activity induced by scanning surfaces with fingertips could be objectively associated with a pleasantness sensation. Previously, we developed a unidimensional measure of pleasantness, the *Pleasant Touch Scale*, quantifying the pleasantness level of 37 different materials. Findings of this study suggested that the sensation of pleasantness was influenced by the average magnitude of the frictional forces brought about by sliding the finger on the surface, and by the surface topography. In the present study, we correlated (i) characteristics of the fluctuations of frictional forces resulting from the interaction between the finger and the surface asperities as well as (ii) the average friction with the sensation of pleasantness.

**Results:**

Eight blindfolded participants tactually explored twelve materials of the *Pleasant Touch Scale* through lateral sliding movements of their index fingertip. During exploration, the normal and tangential interaction force components, *f_N_* and *f_T_*, as well as the fingertip trajectory were measured. The effect of the frictional force on pleasantness sensation was investigated through the analysis of the ratio *f_T_* to *f_N_*, i.e. the net coefficient of kinetic friction, *μ*. The influence of the surface topographies was investigated through analysis of rapid *f_T_* fluctuations in the spatial frequency domain. Results showed that high values of *μ* were anticorrelated with pleasantness. Furthermore, surfaces associated with fluctuations of *f_T_* having higher amplitudes in the low frequency range than in the high one were judged to be less pleasant than the surfaces yielding evenly distributed amplitudes throughout the whole spatial frequency domain.

**Conclusion:**

Characteristics of the frictional force fluctuations and of the net friction taking place during scanning can reliably be correlated with the pleasantness sensation of surfaces.

## Introduction

Most tactile sensations arise from contact with surfaces. When touching a surface, humans perceive some of its physical attributes, such as fine topography, hardness and softness through complex, nonlinear mechanics taking place during sliding and pushing. These mechanics typically result in rapid, but specific oscillations [1]–[5]. On the other hand, a surface can independently elicit a certain degree of pleasantness depending upon those physical attributes. It is believed that a subtype of afferents, described as slowly conducting unmyelinated C-fibers, play a fundamental role in determining the pleasantness character of a stimulus [6]–[9]. These fibers have been identified as C-Tactile (CT) afferents. To date, the CT-fibers have been only identified in hairy skin sites [10]–[12]. However, the everyday experience tells us that surfaces touched with the fingertips can also acquire a character of (un)pleasantness. We therefore wondered whether it was possible to associate some objective physical attributes of surfaces with their pleasantness levels during scanning.

The sensation of pleasantness in touch may be regarded as a *latent* variable rather than an observable variable. Observable variables can be directly quantified and typically generate linear measures expressed with reference to a standard (e.g. the grain size of sandpaper can be measured in micrometers). In contrast, latent variables can only be measured indirectly (e.g. pain, intelligence or pleasantness), generally by using a questionnaire or a set of stimuli, and have no units. In this respect, previous studies attempted to identify which subjective qualities acquired during surface scanning were correlated with the sensation of pleasantness. From a methodological point of view, most of these studies relied on magnitude estimation techniques, or categorical rating methods, to inversely associate a subjective ranking of roughness with a sensation of pleasantness [13]–[16]. Conversely, a subjective ranking of smoothness could be associated with a sensation of pleasantness [17]–[18]. Even if magnitude estimation is an unlimited rating scale method, allowing participants to freely choose a number reflecting their perception of a stimulus [19], it has been determined that magnitude estimation methods, like categorical ranking methods, yield ordinal scores only [20]–[21]. Therefore, both magnitude estimation and categorical ranking methods generate data lacking fundamental scaling properties, including unidimensionality, linearity, specific objectivity, and invariance (*see* Introduction S1 for details on these properties), which precludes objective and quantitative comparison of the measured variable [22]–[23]. These shortcomings eliminate from consideration data analysis through parametric statistical methods. Nevertheless, probabilistic measurement models, such as the Rasch model [24], can be used to determine linear, unidimensional and invariant measures from ordinal scores. In a Rasch analysis, the idea is to statistically model the tendency of each scorer to score more harshly or more leniently than the other scorers with the view to enforce the desirable properties of unidimentionality and linearity of a proper measurement scale. The model is used to rescale and relocate the raw data on common uniform scale. For example, a Rasch analysis can be employed to rank fairly the examinations of a class scored by a group of markers.

In a recent study [25], we used the Rasch model to build a *Pleasant Touch Scale*. This model allowed us, on an objective basis, to construct a scale for use in future studies about the sensation of pleasantness in touch (*see* Introduction S1 for details on the Rasch model). The *Pleasant Touch Scale* considered 37 samples of materials that were classified along a single underlying scale according to their level of pleasantness. The establishment of this scale involved 198 participants and accounted for their individual scoring tendencies. This way, the scale became independent from the fact that the participants scored the same samples differently, that is, with different degrees of leniency (e.g. a less lenient participant had a higher probably to perceive a same material as less pleasant than a more lenient subject). The results of this study showed that materials having an irregular surface topography (e.g. sandpaper) or eliciting high friction during exploration (e.g. wax), had a lower pleasantness level than materials having a more regular surface topography (e.g. paper) or materials being more slippery (e.g. marble). Moreover, an analysis of invariance highlighted the fact that the pleasantness levels of most surfaces were dependent on the participants' fingertip moisture levels. Taken together, the results brought us to formulate the hypothesis that surface topography and frictional properties might strongly be implicated in the sensation of pleasantness during active touch exploration. These indications were objective in the sense that they were based on unidimensional, linear and invariant pleasantness measures of the materials forming the *Pleasant Touch Scale*.

The aim of the present study was to objectively relate a given sample, characterized by its frictional properties, to objective measures of pleasantness (determined through the Rasch model). This study provides evidence that the evaluation of the pleasantness of a texture is correlated with both the net value of friction force and the fluctuations of friction force (reflecting the surface topography, the material with which the sample was made as well as the sample's microstructural properties) created during tactile exploration of the surface.

## Materials and Methods

### Ethic Statement

The present study was approved by the Biomedical Ethical Commission of the Faculty of Medicine of the Université Catholique de Louvain, Belgium (2010/07JUI/174, Belgian registration number: 40320108947). Participants provided their written informed consent to participate in this study. This consent procedure was approved by the ethics committee.

### Participants

We enrolled eight healthy, right handed participants (5 males; age range 23–32 years).

### Apparatus

**Figure 1:**
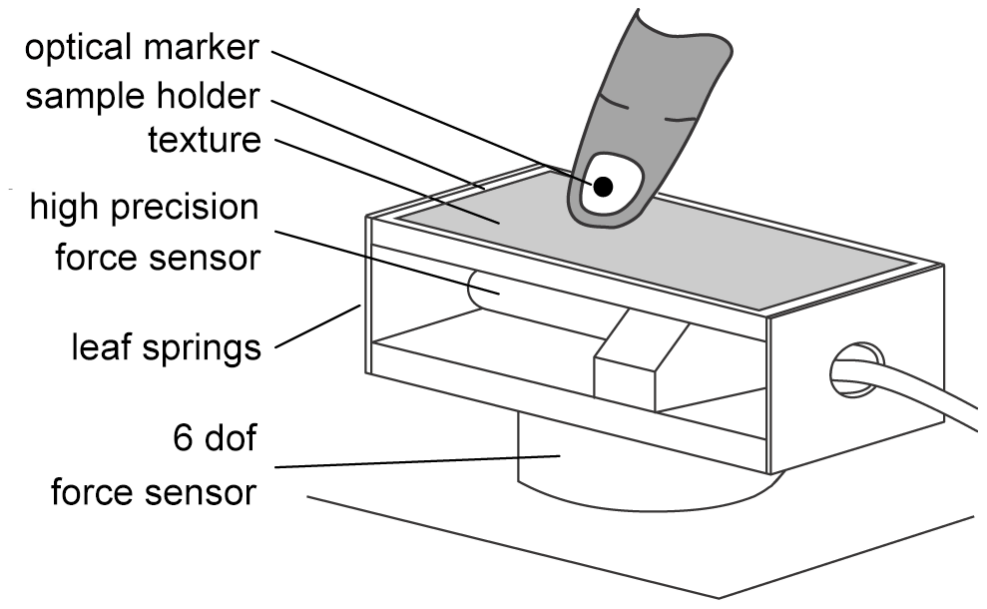
Illustration of the experimental device.

The measurement apparatus (Figure 1) included a sample holder rigidly connected to a high-resolution piezoelectric force sensor (9217a, Kistler Instrumente AG, Winterthur, Switzerland) connected to a charge amplifier (5015A, Kistler Instrumente AG, Winterthur, Switzerland). The piezoelectric force sensor was dedicated to the measurement of the fluctuations of the tangential component of the interaction force, that is, the friction force. This force sensor combines high rigidity with high sensitivity allowing for a 500 Hz exploitable frequency bandwidth and a theoretical noise floor as low as 10 µN. Two parallel leaf springs provided a high rigidity support for the sample holder in the normal and radial directions, and optimal transmission of the interaction force in the lateral direction. The entire structure was connected to a six-axis, strain-gauge force-torque sensor (Mini 40, ATI Industrial Automation, Inc., Apex, NC, USA) that allowed us to gain access to the complete interaction force vector in the low frequencies and with a resolution of 20 mN. The finger position was measured by an optical motion tracking system (Optotrak, Northern Digital Inc., Waterloo, Ontario, Canada) that located a light-emitting fiducial marker attached to the scanning finger nail at a rate of 400 Hz. Acquisition of sensor signals have been made using a 12 bits analog to digital converter at a sampling rate of 20 kHz.

The participants' fingertip moisture levels were evaluated using the Corneometer® CM 825 (CK electronic GmbH, Köln, Germany) which gave readings on a scale ranging from 0 to 120 arbitrary units (a.u.), where lower values represent lower moisture levels.

### Stimuli

Twelve surfaces of the *Pleasant Touch Scale* [25] were selected for this study. These surfaces were chosen because they range from the most unpleasant (sandpaper, P24) to the most pleasant (paper, 160 g/mm^2^) surface of the scale. Each of these samples was bonded to an aluminum plate facilitating their mounting on the measurement device.

The pleasantness levels of the samples, expressed in logits, are listed in Table 1. In the Rasch model, a logit is the unit on the measurement scale. This unit accounts for the transformation of ordinal scores into log odds ratios on the common measurement scale operated by the Rasch analysis (please refer to Introduction S1 for detail). Lower logit values indicate less pleasant samples.

**Table 1:** Twelve materials of the *Pleasant Touch Scale* [25] ordered according to their pleasantness level.

Material	Pleasantness [logit] (from[25])	SE [logit] (from [25])	Total Score [-] (this study)
Sandpaper (P24)	−4.47	0.48	0.00
Sponge	−4.19	0.40	0.00
Latex	−1.88	0.17	4.00
Wax	−1.73	0.17	5.00
Linen	−1.12	0.14	3.00
Wood	−0.64	0.18	9.00
Plastic	0.39	0.15	11.00
Aluminum	0.70	0.17	10.00
Tile	0.81	0.17	13.00
Tights	1.07	0.13	10.00
Velvet	1.74	0.12	10.00
Paper (160 g/m^2^)	2.44	0.14	14.00

*SE*: standard error.

### Protocol

Participants washed and dried their hands. The fiducial marker was fixed to the nail of the participants' right index fingers. Participants were blindfolded and the moisture level of their right index fingertip was measured using the Corneometer® CM 825. The materials were mounted by the experimenter on the measurement device in a randomized order. For each trial, the participants were instructed to position their right index fingertip just above the selected material. On cue, they explored the sample through a lateral sliding movement (from left to right) with a spontaneous exploration force and speed. The participants explored each sample through ten successive sliding movements. During each exploration, the high-frequency fluctuations of tangential force component were recorded, along with the net interaction force, and the fingertip position. After each exploration, the participants were asked to rate the pleasantness of the samples on the basis of a 3-level scale as very pleasant (scored 2), pleasant (scored 1), or unpleasant (scored 0). For each sample, the fingertip moisture level was again recorded immediately after the last exploration trial.

### Data processing

The pleasantness scores were summed, leading to a total score per sample which reflected the overall pleasantness level evaluated by the eight subjects. These total ordinal scores are indicated in Table 1.

All analyses focused on 20 mm (i.e. between 40 and 60 mm of each material) of the active steady-state fingertip slip phase. During this phase, force data were numerically low-pass filtered (butterworth 4^th^ order filter) at 800 Hz and the fingertip position signal was differentiated with respect to time to estimate the exploration velocity. The software package Matlab® (version 7.10) was used to process force as well as fingertip position data.

We firstly computed the mean velocity, *v*, the mean tangential force component, *f_T_*, as well as the mean normal force component, *f_N_*, per sample exploration and per participant. The values of the dynamic coefficient of friction, *μ*, were determined by dividing *f_T_* (of each sample exploration of each participant) by *f_N_* (of each sample exploration of each participant). Secondly, we computed the average values for all parameters over the ten explorations. To investigate the effect of surface topography, the analysis focused on the rapid fluctuations of the friction force, i.e. *f_T_*. Past virtual reality studies have shown that participants can identify complex textured surfaces on the basis of the tangential skin displacement only [5]. Since the finger oscillations resulting from scanning a surface depend on the surface's topography, it can be hypothesized that the fluctuations of the tangential force provide key information regarding the nature of the scanned surface. In the present study, the raw measurements of friction force were sampled in the temporal domain. Yet, we experience surfaces as spatially stable objects and not as time-encoded signals. In the aforementioned study [5], we showed that while people can identify surfaces based on spatially-encoded friction force fluctuations they are unable to do so when the same forces are encoded temporally.

**Figure 2:**
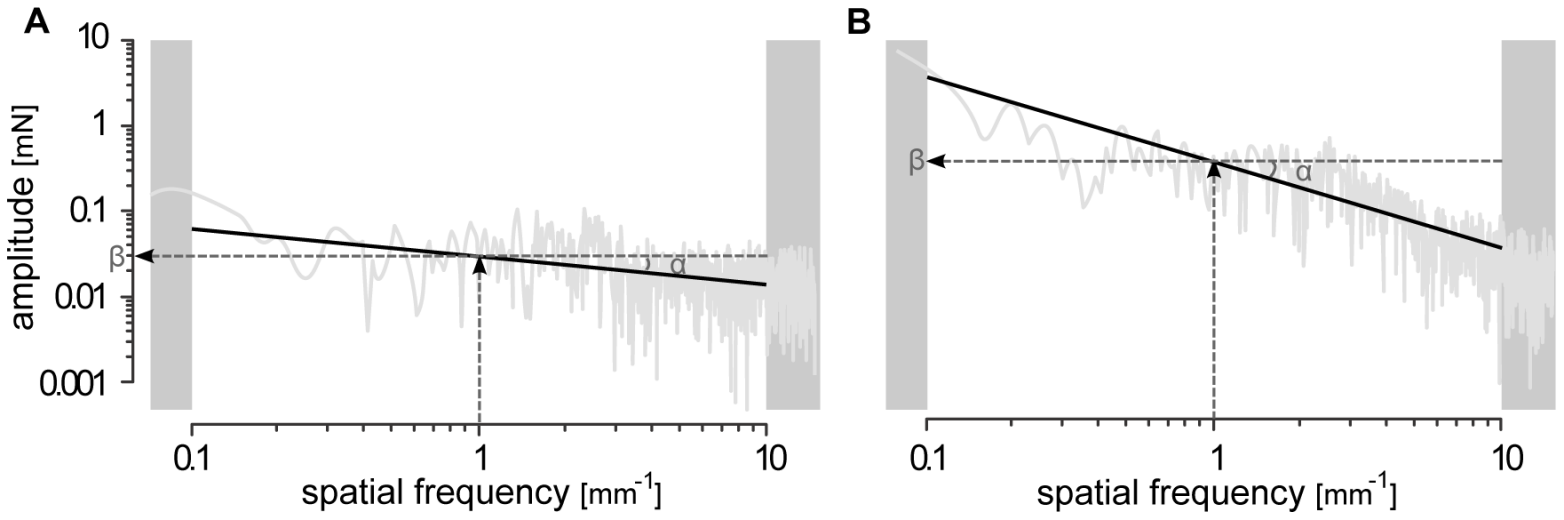
Illustration of rapid force fluctuations in terms of spatial frequencies. Rapid force fluctuations for *paper* (panel A) and for *sandpaper* (panel B). A power function was fitted to the spectrum of each sample.

Owing to finger biotribology, the transformation between surface topography and spatially encoded signals is of non-stationary nature. Nevertheless, an averaging analysis revealed regularities that could be represented by two parameters, *α* and *β*, that encode these regularities: a frequency decay and a scale that are characteristic of particular combinations of materials and surface topographies, roughly in a 1/f fashion as can be expected of mechanical processes [26]. To account for these observations, the raw friction force data of each participant and each exploration was resampled with respect to space, using the corresponding fingertip positions. The change of variable makes the signal insensitive to small variation of velocity across the exploration that would otherwise have affected the temporal frequency content. Following the analysis detailed in [5], as many as 40 000 equally spaced force samples over a 20 mm extent could be produced, which according to the Nyquist sampling theorem could account for 1 µm surface details. A Fast Fourier Transform analysis was then performed on the re-sampled signal to obtain a spatial spectrum. The rapid force fluctuations for each surface could then be analyzed in terms of spatial frequencies. The analysis focused on spatial frequencies situated in the range 0.1 mm^−1^ to 10 mm^−1^. Preliminary inspection of the spectrum confirms the findings of Wiertlewski et al. [26]. Indeed, the spectrum content of the fluctuation of the tangential force for each texture follows an inverse power law. A power function, corresponding to straight line in log-log coordinates, was fitted to the spectrum of each sample to quantify the decay of the value of the friction force with respect to spatial frequency, 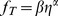
where *η* represents the spatial frequency, *α*<0 the slope of the regression line and *β* its offset. Low values of the decay coefficient, *α*, express the fact that the underlying signal has a tendency to be distributed in the low and high spatial frequencies with similar amplitudes. High values of *β* correspond to the overall strength of the vibrations. The values of *α* and *β* were estimated for each sample and each participant, except for one of them who scanned the samples too quickly to be reliably processed. Figure 2 illustrates the here above described analysis for the most pleasant material of the *Pleasant Touch Scale* (i.e. paper) (Figure 2A) as well as for the most unpleasant material (i.e. sandpaper) (Figure 2B).

### Statistical analyses

A Spearman correlation analysis was conducted to check whether the total ordinal pleasantness scores of each material based on the eight subjects of this study were similar to the sample pleasantness measures defined in [25].

To investigate whether the participants' fingertip moisture levels remained constant during exploration of each surface, a Repeated Measure Analyses of Variance (RM-ANOVA) was conducted, where the “surfaces” and the “time” were defined as “within-participant factors” and the two fingertip moisture levels measured per surface exploration as “within participant variables”. Further, RM-ANOVAs were conducted to determine whether the exploration kinematics, friction and surface topography changed significantly according to the surface being explored. For each of these RM-ANOVAs, the “surfaces” were defined as a “within-participant factor” and “within-participant variables” were respectively *v*, *f_N_*, *f_T_*, *μ*, *α* and *β*.

The correlations between the surfaces' *pleasantness* levels (dependent variable) and respectively *v, f_N_*, *f_T_*, *μ*, *α* and *β* (independent variables) were estimated using a Spearman correlation analyses. The non-parametric coefficient has been used as not all variables were normally distributed.

For all above described analyses, effects were considered significant for p<0.05.

A Principal Component Analysis (PCA) was performed to establish the relationship between measured variables and the materials' *pleasantness* levels. This type of analysis aims at the determination of a small set of factors that captures a complete set of possibly correlated observations. Such analysis is preferably performed in several steps. In a first step, the Kaiser-Meyer-Olkin criterion was used to measure the sampling adequacy. Its value varies between 0 and 1 and should be higher than 0.5. Furthermore, the Bartlett's test of sphericity was used to assess whether the initial dataset was suitable for PCA, i.e. the test of the null hypothesis that the original correlation matrix is an identity matrix, since a PCA can only be conducted if there is correlation between the analyzed variables. Then, the Kaiser criterion was used to define the number of factors to be extracted. This method is based on the principle of retaining only the factors associated with eigenvalues greater than one (i.e. the total variance of each factor). Thereafter, the oblique rotation, namely the direct oblimin rotation, was performed to maximize the loading of each variable on one of the extracted factors, while minimizing the loading on all other factors. In contrast with orthogonal rotation methods, the oblique rotation allows the factors to be correlated [27]. All statistical analyses were conducted using IBM® SPSS® Statistics (version 20).

## Results

**Figure 3:**
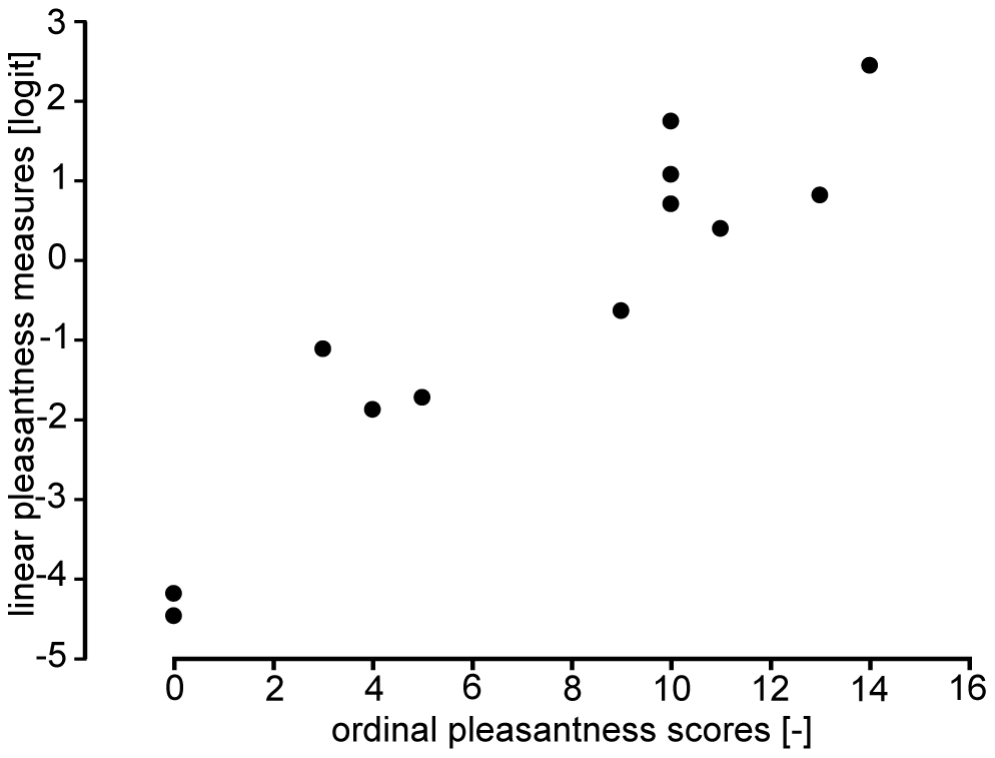
Correlation between the ordinal pleasantness scores of the present study and the unidimensional linear pleasantness measures determined in [25]. Each point represents one sample of material.

The total ordinal pleasantness scores of the eight subjects were highly correlated with the linear, unidimensional pleasantness measures determined in [25] (ρ = 0.9; p<0.001, see Figure 3), validating the pleasantness scale established for the present study. These measures were thus employed in the subsequent statistical analyses.

The participants' fingertip moisture levels did not vary significantly between first and last exploration of each material (F_(1,7)_ = 1.70; p = 0.23). Mean fingertip moisture levels ranged from 32±16 (sandpaper) to 43±19 (linen) arbitrary units (a.u.).

Participants were free to choose their own exploration strategy. The range and the mean values of their spontaneous exploration kinematics are indicated in Table 2.

**Table 2:** Characterization of studied variables.

Variable	n	Mean (mean±std)	Range (min-max)	RM-ANOVA		Correlation	
*v*	8	104.0±56.4 mm/s	42.0–321.0 mm/s	F_(11,77)_ = 1.66	P = 0.99	ρ = 0.16	p = 0.130
*f_N_*	8	0.7±0.3 N	0.2–1.6 N	F_(11,77)_ = 1.82	p = 0.65	ρ = 0.14	p = 0.187
*f_T_*	8	0.5±0.3 N	0.1–1.5 N	F_(11,77)_ = 5.99	p<0.001	ρ = −0.45	p<0.001
*μ*	8	0.7±0.4 N	0.3–1.6	F_(11,77)_ = 38.80	p<0.001	ρ = −0.65	p<0.001
α	7	−0.8±0.2	−1.2–−0.4	F_(11,66)_ = 9.13	p<0.001	ρ = 0.46	p<0.001
*log_10_β*	7	−3.8±0.5	−4.6–−2.5	F_(11,66)_ = 33.09	p<0.001	ρ = −0.80	p<0.001

*RM-ANOVA*: repeated-measure analysis of variance; *Correlation:* spearman correlation between each variable and the materials' *pleasantness* levels; *v*: exploration velocity; *f_N_*: normal force component; *f_T_*: tangential force component; *μ*: dynamic coefficient of friction; *α*: decay coefficient of friction-induced vibrations in the spatial domain; *β*: the offset of friction-induced vibrations in the spatial domain.

We first wondered whether participants spontaneously adapted their exploration kinematics according to the surfaces being explored. The results of the corresponding RM-ANOVAs showed that participants neither significantly adapted their exploration velocity, *v*, nor significantly modified the normal force, *f_N_*, according to the surface being scanned (Table 2). Each participant adopted a preferred exploration strategy which was by-and-large the same for all surfaces being explored. The results of the Spearman correlation analysis were in line with this observation. Neither *v* nor *f_N_* was significantly correlated with the *pleasantness* levels of the surfaces (Table 2).

**Figure 4:**
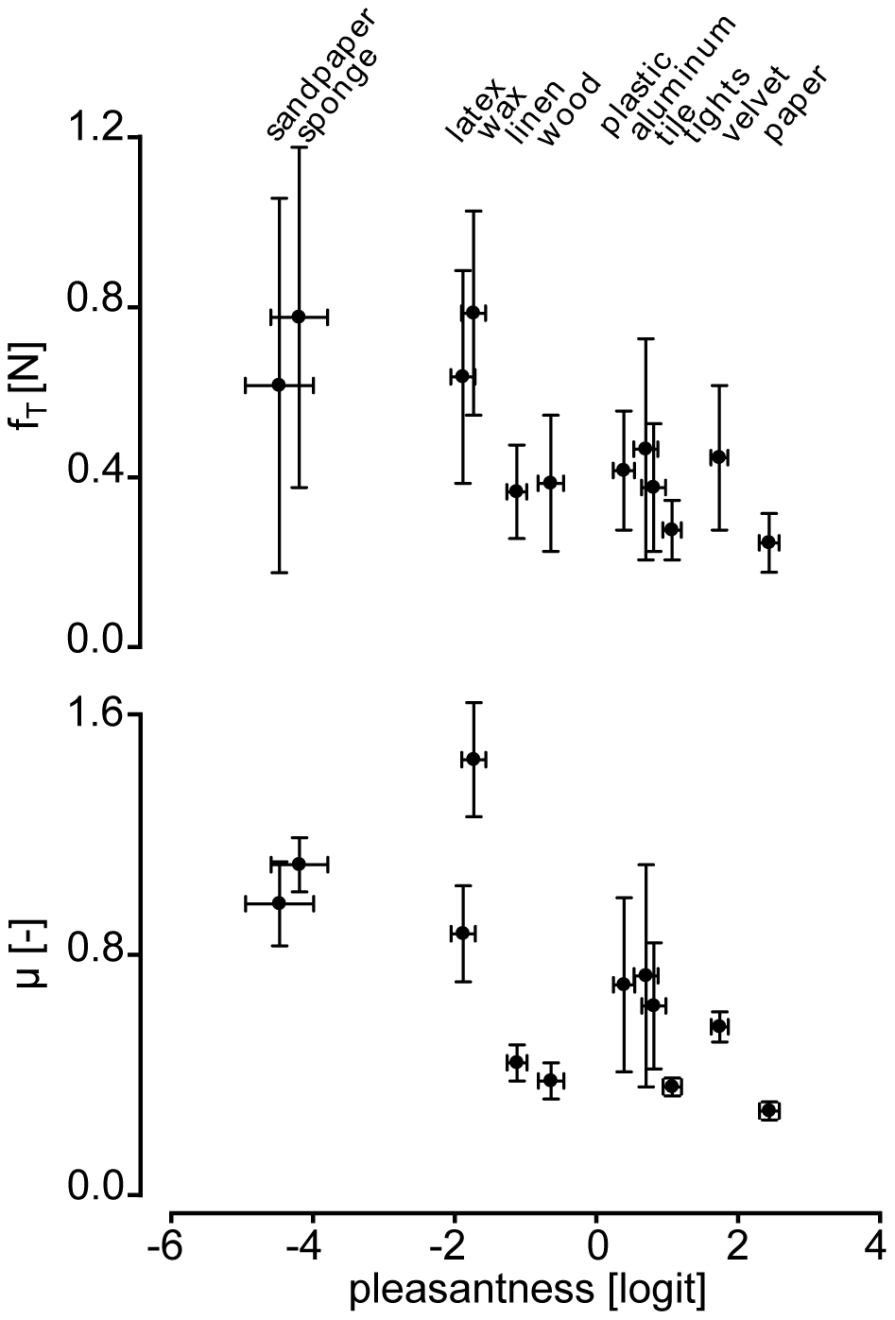
Illustration of correlations between (i) *f_T_* - *pleasantness* and (ii) *μ* - *pleasantness*. Mean±std tangential force component, *f_T_*, (top) and the mean±std dynamic coefficient of friction, *μ*, (bottom) variations according to the material *pleasantness* levels±standard error being explored.

**Figure 5:**
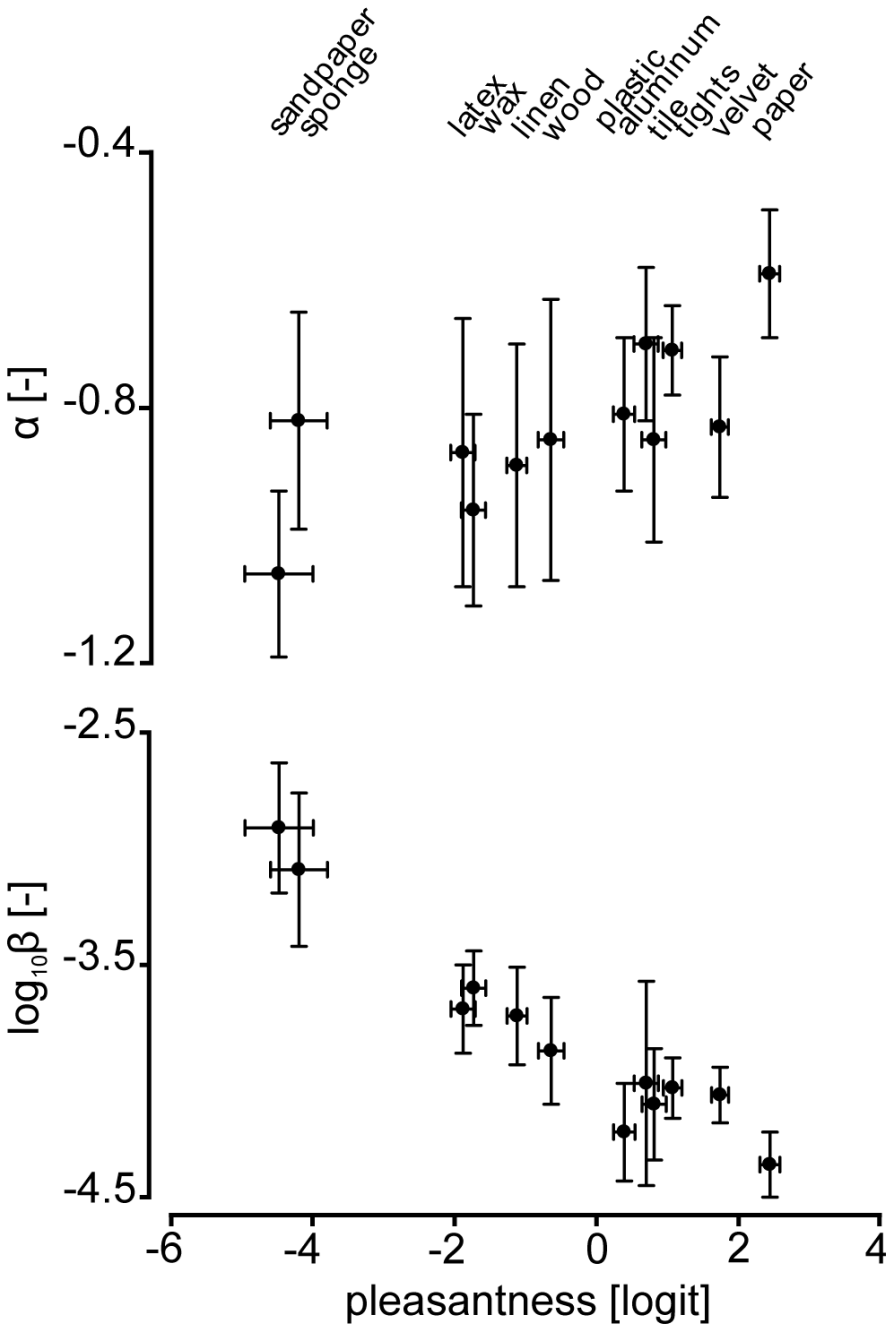
Illustration of correlations between (i) *α* - *pleasantness* and (ii) *β* - *pleasantness*. Illustration of the mean±std values of the decay coefficient, *α*, (top) and the mean±std values of the offset, *β*, (bottom) variations according to the material *pleasantness* levels±standard error being explored.

In a second step, the investigation focused at determining whether the tangential force, *f_T_*, the friction coefficient, *μ*, and the regression parameters of the spectrum, *α* and *β*, varied significantly according to the surface being explored. The results of the corresponding RM-ANOVAs showed that *f_T_*, *μ*, *α* and *β* varied greatly according to the scanned surface (Figures 4, 5; Table 2). In addition, the results of the Spearman correlation analysis highlighted that these same variables were significantly correlated with the *pleasantness* levels of the surfaces (Figures 4, 5; Table 2). Variables reflecting friction occurring during surface exploration (i.e. *f_T_* and *μ*) were negatively correlated with the *pleasantness* levels, suggesting that high friction is associated with a low level of *pleasantness*. The character of the motion-induced vibration was also correlated with *pleasantness*. The decay coefficient, *α*, was positively correlated with *pleasantness* and the offset, *β*, was negatively correlated with the surface *pleasantness* level. In other words, surfaces are perceived to be more pleasant if the spectrum of the friction force fluctuations is evenly distributed in the low and high spatial frequencies and the resulting vibration strength is low. Figure 2 illustrates this observation. Indeed, in contrast to the friction induced vibrations of ‘*sandpaper*’ (Figure 2B), those induced by ‘*paper*’ (Figure 2A) are evenly distributed in the low and high frequencies.

**Figure 6:**
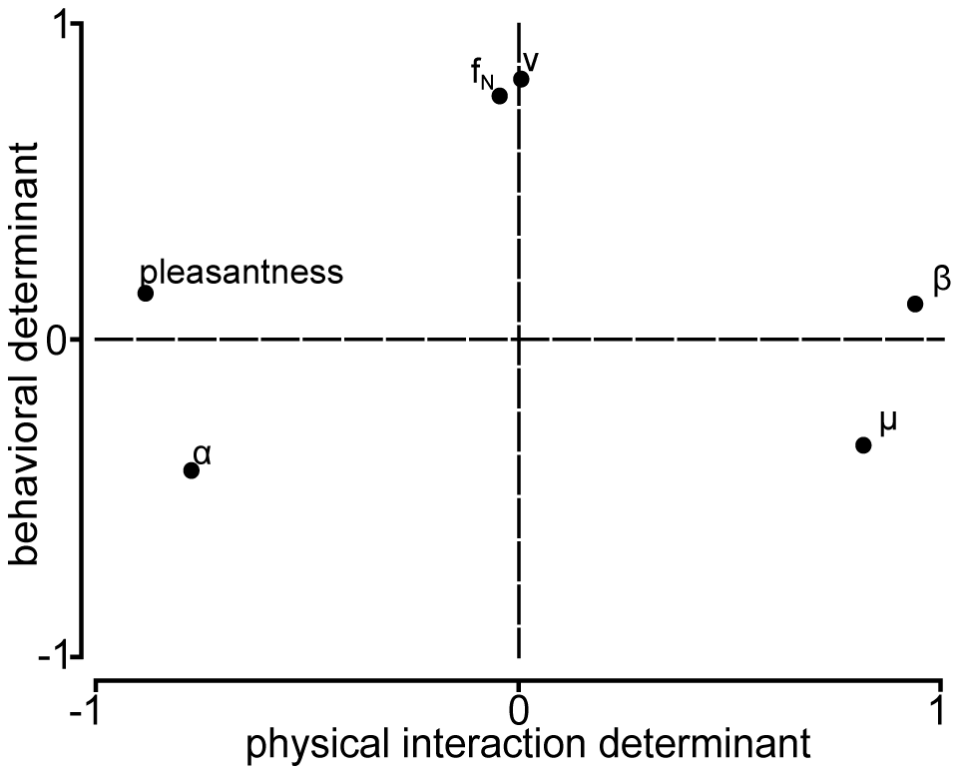
Illustration of the Principal Component Analysis. Representation of the two first components summarizing our data set. The co-ordinates of the components along each axis represent the strength of relationship between that variable and each component.

A final analysis step was performed in order to assess whether some variables could be grouped into independent factors interacting with the level of *pleasantness*. This investigation was carried out through a PCA on six variables, namely: *v*, *f_N_*, *μ*, *α*, *β* and surfaces' *pleasantness* levels. Non-normally distributed variables were logarithmically transformed for this analysis. Our data set was appropriate for PCA analysis since the Kaiser-Meyer-Olkin measure of sampling adequacy was 0.6 and the Bartlett's test of sphericity was highly significant (<0.001). The PCA highlighted that two subsets of variables could be extracted, explaining 74.8% of the total variance of the data set. Table 3 collects the respective factor loadings after rotation. A factor loading corresponds to the coordinate of each variable along a factor and indicates therefore the correlation level between a factor and a variable. In Figure 6, each factor is represented by a single axis, and the coordinates indicate the strength of the relationship, i.e. the correlation between each variable and both factors. Figure 6 shows that *α*, *β*, *μ* and the surfaces' *pleasantness* levels form a first subset of variables (or factor) (explaining 48.5% of the variance), termed ‘*physical interaction determinant*’. The *f_N_* loaded significantly with *v* on the second factor (explaining 26.3% of the variance), termed ‘*behavioral determinant*’.

**Table 3:** Factor loadings of each variable as determined by the Principal Component Analysis.

Variable	Physical interaction determinant	Behavioral determinant
*pleasantness*	−0.88	0.15
*f_N_*	−0.04	0.77
*β*	0.94	0.11
*α*	−0.77	−0.41
*μ*	0.81	−0.33
*v*	0.01	0.82

*pleasantness*: materials' pleasantness levels; *f_N_*: normal force component; *β*: the magnitude of friction-induced vibrations in the spatial domain; *α*: decay coefficient of friction-induced vibrations in the spatial domain; *μ*: dynamic coefficient of friction; *v*: exploration velocity.

## Discussion

We have described some physical factors that are correlated with the sensation of pleasantness during active surface exploration with the fingertip. These factors are the average coefficient of friction (*μ*), the average magnitude of the tangential interaction force component (*f_T_*) as well as the offset (*β*) and the decay coefficient (*α*) of friction-induced vibrations in the spatial domain.

In this and our previous study [25], the participants explored the different samples through standardized active lateral sliding movements of their index fingertip over the surfaces. The rationale behind the choice of *lateral* and *active* sliding movements was twofold. Firstly, several previous studies, e.g. [28], showed the dominance of medial-lateral sliding movements during texture discrimination. Secondly, according to the duplex theory of roughness perception, coarse textures are mediated by somatotopically encoded tactile inputs while fine textures are mediated by vibrations [1]; [29]–[32]. Consequently, fine textures can only be discriminated when there is relative sliding between the skin and the sample [30]. In the present study, the samples spanned a wide range of different microstructures and materials. The participants were instructed to employ laterally sliding exploration movements that are efficient for all combinations of these features. In future studies, it could be interesting to study the effect of movement strategies and anatomical regions on the sensation of tactile pleasantness (e.g scratching with the nail, employing the volar region of the wrist, the feet and so on).

Our analysis revealed that unpleasant surfaces elicited high friction when a fingertip was actively slipped on them. In a past study, Gwosdow et al. [33] investigated pleasantness sensations given by various fabrics rubbed across the participants' inner forearms. The authors found that higher moisture levels caused an increase of friction at the skin-fabric interface that was associated with a decrease in pleasantness. This finding pointed to a relationship between friction and pleasantness, but it remained subjective and statistically unsupported. A more recent study [17] indicated that the subjective sensation of ‘stickiness’ was positively correlated with a subjective rating of pleasantness during surface exploration with the fingertip. It can be hypothesized that a subjective quality of ‘stickiness’ is closely linked to particular frictional behaviors taking place during exploration. The results of our study run counter to this finding. This discrepancy might be explained by the fact that, in contrast to participants of our study, participants of the study of Chen et al. [17] might have had very high fingertip moisture levels. Indeed, Tomlinson et al. [34] highlighted that very high levels of fingertip moisture decreases the dynamic coefficient of friction during surface exploring, which in turn increases the materials' pleasantness levels. In future studies, it could be of interest to investigate whether manipulation of friction of an object, e.g. by artificially changing the fingertip moisture level of a participant exploring the object, changes the participant's pleasantness perception.

The friction-induced vibration also imparts pleasantness estimation. Our analysis of the fluctuation of the tangential force in the spatial domain suggests that unpleasant materials have an overall higher amplitude of vibration as well as a distribution of frequency that favors the low part of the spectrum. Friction-induced oscillations depend on the surface topography. Past studies indicated that the subjective rating of roughness was inversely correlated to the sensation of pleasantness during active surface exploration [13]–[16], which indicates a link between the surfaces' topographies and their respective pleasantness ratings. Ekman et al. [14] investigated the correlation between the subjective sensation of smoothness and roughness with the finger-surface coefficients of static friction. Roughness was correlated with higher friction and smoothness was correlated with lower friction. The existence of a link between smoothness and surface preference was also proposed in this study, which indirectly suggests a link between frictional properties and preferences.

To our knowledge, however, no previous study attempted to correlate objective physical measurements with objective pleasantness measures. The present study took advantage of the direct measurement of the friction-induced mechanical interactions rather than the indirect effect of the topography of a sample. The actual friction-induced force fluctuations are an accurate reflection of the mechanical stimulus available to the brain, while surface topography, is entangled with several other factors that include the material microstructure, its porosity, the average friction, the constituting materials, the presence of water and other complex tribological factors that are at the root of the highly nonlinear transformation from microgeometry to interfacial force fluctuations. Other studies investigating in roughness perception of fine textures have shown that such perception relies on a vibratory signals [30]–[32] transmitted through the Pacinian Channels (PC) [29]; [31]. Furthermore, Bensmaia et al. [29] found that this channel is “*capable of conveying sufficient information to mediate the identification and discrimination of fine textures*” [29]. As we highlighted that vibrations induced by surface exploration with fingertip have an impact on the pleasantness perception of the scanned surface, it might be hypothesized that PCs are implicated in transmitting crucial information for pleasantness perception.

In agreement with the study of Smith and Scott [35], the present study supported the hypothesis that participants prefer certain exploration strategies, independently from the surface being explored. All participants *spontaneously* used exploration velocities (*v*) and average normal interaction forces (*f_N_*) which were not significantly different according to the material being explored. This result strengthens the hypothesis that participants rate pleasantness levels through comparisons of the characteristics of the frictional force fluctuations and average friction while keeping behaviorally-controlled parameters such as applied normal force and movement speed *intuitively* invariant. It has been suggested that Slowly Adapting type I (SAI) and Fast Adapting type I (FAI) afferents “*provide the neural basis for peripheral signals of tangential force magnitude*” [36]. Interestingly, Wheat et al. [36] showed that the sensitivities of SAI and FAI afferents to tangential force levels were independent of the normal force levels, which could enable the brain to extract information about the tangential force without cross-talk from the normal force [36]. We have highlighted that the *pleasantness* level of a surface is (i) highly dependent on the characteristics of friction force fluctuations as well as average friction induced by the surface and (ii) that participants *spontaneously* keep their normal interaction force used to explore the surface invariant. As a consequence, it might be hypothesized that SAI and FAI afferents are implicated in the transmission of important information regarding the pleasantness level of an explored surface.

The PCA conducted in this study highlighted that the factor ‘*physical interaction determinant’* explained approximately 49% of our data set's variance. This factor regroups the variables materials' *pleasantness* levels, *μ*, *α* and *β*, where *μ* and *β* are negatively and *α* positively correlated with the materials' *pleasantness* levels. It has, however, to be noted that the effect of several surface properties (e.g. compliance and temperature) on the *pleasantness* levels could not be investigated in this study. Nevertheless, increasing the number of analyzed variables could have increased the amount of explained variance of the factor ‘*physical interaction determinant*’. Consequently, this could have provided additional insight into variables having an impact on pleasantness perception. Finally, it is worth noting that a past study highlighted a link between the temperature of stimulus and its affective perception at level of the hand [37]. Consequently, it could be of interest to objectively investigate the effect of temperature on the pleasantness perception of a stimulus explored with the fingertip, in addition to *μ*, *α* and *β*. Such study could have the effect of increasing the amount of explained variance of the component ‘*physical interaction determinant*’.

## Supporting Information

Introduction S1 Introduction to the Rasch model.
(DOCX)
