# Supplementary material for: Physical Factors Influencing Pleasant Touch during Tactile Exploration
Source: PLoS One. 2013 Nov 14;8(11):e79085. doi: 10.1371/journal.pone.0079085 (PMC3828339; doi:10.1371/journal.pone.0079085)
Supplement: Introduction S1 — Introduction to the Rasch model. (DOCX) [file pone.0079085.s001.docx]

**Supporting Information – Introduction S1**

*The Rasch Model*

Observable variables can directly be measured and generally generate *linear measures* (e.g. the dimension of an object can directly be determined through the use of a meter). In contrast, latent variables (e.g. pain, intelligence or pleasantness) can only be measured indirectly, generally by using a questionnaire or a set of stimuli. Such measurement provides *ordinal scores* without unit. Ordinal scores come with severe shortcomings, precluding the *objective measurement^[[1]](#footnote-1)^* of a variable [1-5]. Firstly, ordinal scores are not necessarily *linear* (i.e. the measurement unit is not constant throughout the measurement, [6]). For instance, equal distances (e.g. from 0 to 1 and from 1 to 2) may not reflect the same amount of the measured variable. Secondly, ordinal scores are not necessarily *unidimensional* (i.e. the measurement reflects not only one attribute of an object, [7]). As a result, adding individual ordinal scores may result in a total score that is meaningless. Thirdly, ordinal scores are not necessarily *objective* (i.e. not sample-free and item-free measurements [7]). For example, the measurement of the performance of persons through methods generating ordinal data is item dependent (the ranking of students' works, marked by different graders, depends on the graders). Conversely, the evaluation of scales is sample dependent [8]. Lastly, ordinal scores are not necessarily *invariant* under changes of the populations employed to generate the data (e.g. systematic differences between males/females) [7].

Probabilistic measurement models, such as the Rasch model [2], can be used to establish linear, unidimensional, and invariant scales from ordinal scores by locating subjects and items along a single underlying scale. This model enables the objective measurement of latent variables, and thus opens the way to quantitative analyses.

The Rasch model can be applied to the analysis of polytomous data, that is data arising from more than two response categories, e.g. “very pleasant/pleasant/unpleasant” [9].

However, to be able to construct a linear, unidimensional and invariant measurement scale from such ordinal data, the data to model fit must be tested. A Rasch analysis of polytomous data, tests for the probability that a subject “*n*” will choose category “*x*” for item “*i*”. This probability should be influenced only by the subject’s ability to score items (*β_n_*), the item’s difficulty to be scored (*δ_i_*), and the threshold (*τ_ij_*) between two successive response categories [9]:

where, *m_i_* represents the number of categories of item i and *τ_ij_* corresponds to the j^th^ threshold of item i (arbitrarily, *τ_i0_* = 0 so that *e^(^*^0^*^(βn-δi)-^*^0^*^)^* = 1). On this basis, the expected pattern of response to a set of items can be calculated [10]. The data to model fit can then be verified by comparing the expected responses from the model and the observations. The invariance of the scale is thereafter checked. If the data to model fit is verified, then the Rasch model has reliably transformed the ordinal data (i.e. the originally collected data) into linear, unidimensional and invariant measures [10]. These measures are expressed in logits, the unit of measurement after transformation of the ordinal raw scores into log odds ratios (i.e. ratio between the probability of observing the event and the probability of not observing the event) on a common interval scale [7]. One logit is the distance along the line of the variable that increases the odds of observing the event by a factor of 2.71, the value of *e*, the base of the natural logarithm [11]. For example, if the probability of observing the event “A” is 5/100 = 0.05, then the probability of not observing the event equals 1 – 0.05 = 0.95. In this case, the odds ratio = 0.05/095 = 0.05 and the log odds ratio = ln(0.05) = -3. The probability of observing the event “A+1” = 0.12, whereas the probability of not observing the event “A+1” equals 1 – 0.12 = 0.88. Therefore, the odds of observing the event “A+1” = 0.12/0.88 = 0.14 = 0.05*2.71.

We used this model in a past study to elaborate a *Pleasant Touch Scale* [12], which classified materials according to their pleasantness levels as well as participants along a single underlying scale. The scale invariance analysis allowed us to verify that the subjects’ fingertip moisture levels had an impact on the materials pleasantness levels. This finding points thus to the fact that friction resulting from sliding exploration of the samples by the fingertips, might have an impact on the sensation of pleasantness.

*References*

1 Mellers BA (1983) Evidence against "absolute" scaling. Percept Psychophys 33: 523-526.

2 Rasch G (1960) Probabilistic models for some intelligence and attainment tests. Chicago: Mesa Press 199 p.

3 Tesio L, Simone A, Bernardinello M (2007) Rehabilitation and outcome measurement: where is Rasch analysis-going? Eura Medicophys, 43:417-426.

4 Thurstone LL (1928) Attitudes can be measured. American Journal of Sociology, 33: 529-554.

5 Wills CE, Moore CF (1994) A controversy in scaling of subjective states: magnitude estimation versus category rating methods. Res Nurs Health 17: 231-237.

6 Penta M, Arnould C, Decruynaere C (2005) Développer et interpréter une échelle de mesure. Applications du modèle de Rasch. Sprimont: Mardaga 186 p.

7 Bond TG, Fox CM (2001) Applying The Rasch Model: Fundamental Measurement in the Human Sciences. Mahwah, New Jersey: Lawrence Erlbaum Associates 255 p.

8 Hobart J, Cano S (2009) Improving the evaluation of therapeutic interventions in multiple sclerosis: the role of new psychometric methods. Health Technol Assess, 13: 1-177.

9 Wright BD, Masters GN (1982) Rating Scale Analysis. Rasch Measurement. Chicago: Mesa Press 206 p.

10 Tennant A, McKenna SP, Hagell P (2004) Application of Rasch analysis in the development and application of quality of life instruments. Value Health, 7: S22-S26.

11 Wright BD, Stone MH (1979) Best Test Design. Rasch Measurement. Chicago: Mesa Press 222 p.

12 Klöcker A, Arnould C, Penta M, Thonnard JL (2012) Rasch-Built Measure of Pleasant Touch through Active Fingertip Exploration. Front Neurorobot 6: 1-9.

1. The Institute for Objective Measurement (IOM, www. Rasch.org) defines objective measurement as “*the repetition of a unit amount that maintains its size, within an allowable range of error, no matter which instrument, intended to measure the variable of interest, is used and no matter who or what relevant person or thing is measured*”. [↑](#footnote-ref-1)
